# Development of LED Package Heat Dissipation Research

**DOI:** 10.3390/mi13020229

**Published:** 2022-01-30

**Authors:** Peisheng Liu, Chenhui She, Lipeng Tan, Pengpeng Xu, Lei Yan

**Affiliations:** Jiangsu Key Laboratory of ASIC Design, School of Information Science and Technology, Nantong University, Nantong 226019, China; 1811310001@stmail.ntu.edu.cn (C.S.); tanlipengdsg@163.com (L.T.); 2110320054@stmail.ntu.edu.cn (P.X.); 2110310042@stmail.ntu.edu.cn (L.Y.)

**Keywords:** LED, heat dissipation, packaging technology, packaging structure

## Abstract

LEDs are widely used in medicine, navigation and landscape lighting. The development of high-power LED is a severe challenge to LED heat dissipation. In this review, packaging technology and packaging structure are reviewed in terms of the thermal performance of LED packaging, and related technologies that promote heat dissipation in LED packaging are introduced. The design of three components to enhance heat dissipation in LED packaging is described: substrate, lens and phosphor layer. By conducting a summary of the technology and structure of the package, the defects of LED package technology and structure are deeply investigated, and the package is prospected. This has reference value for the heat dissipation design of the LED package and helps to improve the design and manufacture of the LED package.

## 1. Introduction

A light-emitting diode (LED) is a solid-state semiconductor device that can directly convert electrical energy into visible light [1]. As the fourth-generation lighting source, it has better energy saving, better environmental protection, higher luminous rate, faster start-up speed and longer service life [2,3]. It can meet the needs of various displays [4], decoration [5], indication [6] and lighting [7,8]. Packaging plays a vital role in semiconductor devices. It provides power, cooling functions and protective layers for the chip. With the development of chip design, packaging and high current characteristics, high-power LED packaging has become an urgent need [9]. The increase in the power of the package affects the heat dissipation performance of the device, which also influences the life of the LED and its luminous performance [10]. Therefore, the heat dissipation of LED packaging is currently a hot research topic [11]. This article mainly summarizes LED packaging technology in heat dissipation, analyzes the important components in the path of heat dissipation of package and introduces the improvement of the structure of each component in terms of heat dissipation performance in details.

## 2. Packaging Technology

Packaging technology is an indispensable part of packaging. The quality of packaging technology determines the reliability of the package and the service life of the device. In order to meet the increasing demand for lighting, R & D and technology of larger output LEDs have been extensively developed, and the packaging technology of LEDs has evolved from single-chip packaging to multi-chip packaging [12]. Single-chip packages include surface mounts and power packages [13]. These packages are only appropriate for low-power applications. Similarly, dual inline-pin packages (DIPs) [14] and quad flat package (QFP) [15] have also evolved. DIP is welded by perforation so that the package is bulky and inconvenient to operate. QFP makes the pin-to-chip distance insignificant and is mostly used for large-scale integration. Chip-on-board (COB), wafer level package (WLP) and chip-scale package (CSP) are multi-chip packages. COB assigns chips directly to the board and coats them with a phosphorescent glue [16], which is at a higher packing density [17]. COB, WLP and CSP are more popular LED packages on the market.

With the development of devices toward miniaturization and thinning, the heat dissipation structure and technology of LED packages have also been developed accordingly. The technology of flip-chip LED packaging; through-silicon via (TSV) and 3D packaging; and ultra-thin packaging are all developed under this situation.

### 2.1. Flip-Chip LED Structure

**Figure 1:**
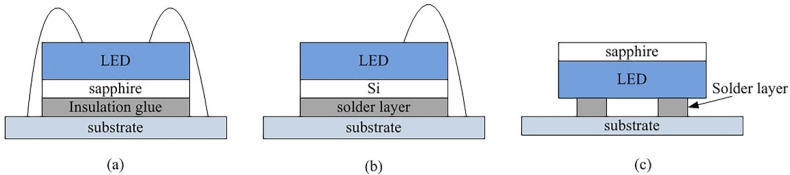
Three high-power LED packaging formats: (**a**) conventional structure, (**b**) vertical LED and (**c**) flip-chip.

Researchers have invented vertical structure and flip-chip structure to improve the problem of easy breakage of gold wire and poor heat dissipation of the conventional package of the LED. The diagram of these three chip architectures is shown in Figure 1.

**Figure 2:**
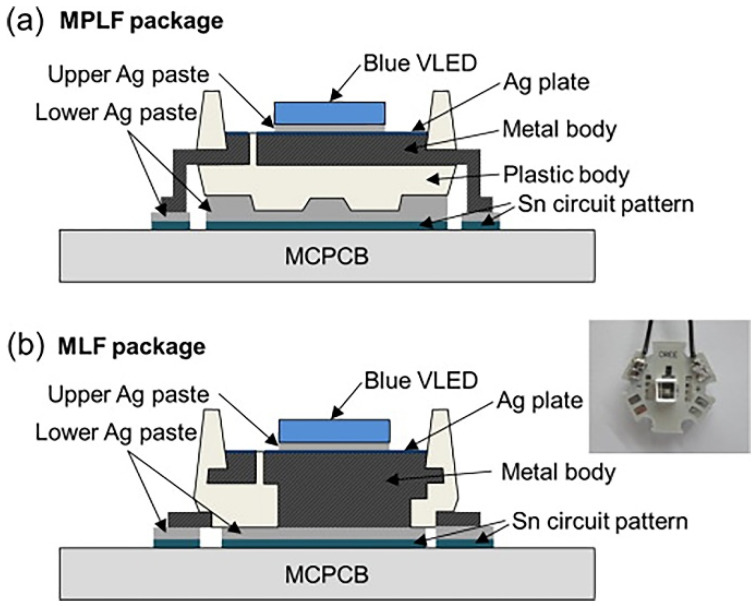
Schematic diagram of blue VLEDs mounted on (**a**) a lead frame with a metal/plastic body (MPLF package) and (**b**) a lead frame with a metal body (MLF package) [21].

The vertical structure [18] is produced from the conventional one. The chip of the conventional sapphire substrate is inverted and bonded on the silicon substrate with better thermal conductivity before the sapphire layer is peeled off. The two electrodes of the vertical LED chip are located on both sides of the LED epitaxial layer. The current through the n-electrode flows vertically through the epitaxial layer, which makes the current flowing laterally very little and avoids local high temperatures. Many studies have been published on this structure to show that vertical LED structures produce better heat dissipation in the LED packages and can improve the convenience of the LED lamp assembly in experiments [19]. Lin et al. [20] proposed using a vertical structure in a high-power LED package to allow high-power LEDs to be efficiently and simply assembled to the lamp socket, which also shortens the distance between the chip and the aluminum plate and significantly improves the reliability of the high-power LED package and its thermal fatigue. Guan et al. [21] investigated the characteristics of GaN-based blue vertical light-emitting diodes (VLEDs) with two different package structures, namely a lead frame with a metal/plastic body (MPLF package) and a lead frame with a metal body (MLF package), under various conditions. Figure 2 shows the structure of the two different VLEDs. The body of the MPLF package is composed of a 2-micrometer-thick silver paste, 200 μm of copper (Cu) and a 400 μm plastic layer, whereas the body of the MLF package is composed of a 2 μm Ag paste and 600 μm of copper. A three-dimensional steady-state device model is used to thermally simulate VLEDs with two different packages, and the authors found that the plastic layer acts as an isolation layer, resulting in higher thermo-mechanical stress in the MPLF packages. Therefore, the MLF-packaged VLEDs have a relatively lower junction temperature and a lower thermal resistance due to the improved heat dissipation.

**Figure 3:**
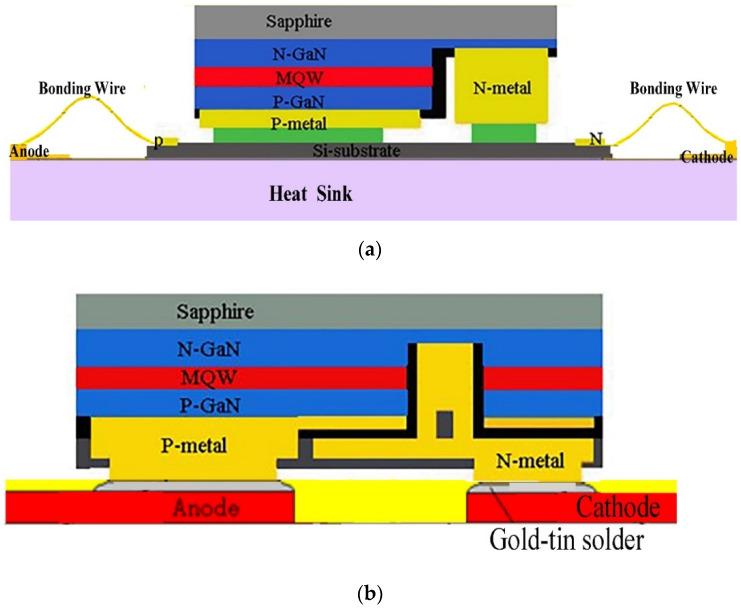
Schematic diagram of wired flip chip package structure (**a**) and wireless flip chip package structure (**b**).

The stripping process of sapphire is difficult in the vertical structure, which restricts the development of industrialization. Therefore, the flip-chip structure LED has gradually received extensive attention from the lighting industry because of its integration, mass production and excellent performance [22]. The flip-chip structure is the opposite of the traditional structure and avoids light absorption by the metal and contact with the conventional chip. Compared with the positive-loading chip, the light-emitting area of the electrode in the flip-chip structure greatly affects the luminous efficiency. Most importantly, heat cannot pass through the sapphire substrate of the chip but transfers directly to the silicon or ceramic substrate with a higher thermal conductivity so that the heat dissipation distance accounts for 1/3 to 1/4 of the vertical LED chip package. However, the wires used in a flip-chip structure were easily broken by the thermal mismatch of various packaging materials from the action of thermal shock. Developing a model that requires no wires to produce flip-chip packages is imminent. As shown in Figure 3a, for the wired flip package, the LED flip chip is fixed to the substrate by using heat conduction assembly glue and electrically connected by gold wires. For the flip chip, the dress LED chip is flipped to the Si substrate by embedding gold balls, and electrodes are prepared on the Si substrate to form the flip chip. The wireless flip chip package, as shown in Figure 3b, consists of horizontal electrode chips plated with Sn or Au-Sn alloy on the electrode contact face that are directly welded to the substrate plated with gold or silver by using eutectic/reflux welding technologies for chip fixing, electrical connection and heat conduction. The wires used in a flip-chip structure were easily broken by the thermal mismatch of various packaging materials from the action of thermal shock. The wireless package is completely free of constraints of the lead and assembly glue. The wireless structure also has excellent electrical and thermal properties [23].

The flip-chip structure can be used in a variety of package formats, including chip-scale packaging (CSP) [24] and wafer-level packaging (WLP) [25]. The CSP package structure is described in detail in the reference [24]. The wafer-level package of a flip-chip LED [25] does not require soldering and can be driven at higher currents. In addition, it has an excellent thermal spread in the vertical direction. However, it is practically difficult to use a flip-chip directly in a wafer-level package. Elger et al. [26] obtained a WLP-LED by attaching a phosphor to the sapphire in a flip-chip followed by a TiO_2_ side coating. Finally, the WLP-LED is mounted directly on the printed circuit board (PCB) of the lighting application. This structure is simple and flexible and presents a lower thermal resistance at a lower cost. 

Although the flip-chip LED package solves the problem of LED lights not switching on due to false soldering, breakage and poor contact of the gold wire, it improves the heat dissipation performance and also causes heat migration and voids in the flip-chip solder layer. The solder layer of the chip is an important element for heat dissipation, electronic conductivity and mechanical support. The degassing of organic solder paste during reflow soldering is the main reason for the formation of voids. An increasing void ratio in the die bond solder layer reduces the efficiency of heat dissipation and contributes to an increase in LED junction temperature [27]. Sn-3.0Ag-0.5Cu (SAC305) solder alloys have lower soldering temperatures and lower costs than gold–tin eutectic solder alloys (Au80Sn20). Consequently, it has been extensively employed as a bonding material for high-power chips [28]. However, the voids generated during reflow soldering and use of SAC305 will affect the heat dissipation and mechanical properties of the LED package. Jiang et al. [24] used CSP flip-chip technology to analyze the effects of the voids on the mechanical and thermal properties of the bonding layers used in the chip-scale packaging of high-power LEDs. When the size of the void increases, the maximum principal stress increases significantly for the same load, and the mechanical properties are reduced. In order to take into account the influence of the location of voids on the mechanical properties of the die-attached solder layer, a single void with a 10% void ratio was placed at different locations in the solder layer of the CSP model. The authors showed that the solder layer with corners close to the outer boundary of the pad and close to the loading position is subjected to the highest stress for a given load, whereas the solder layer located in the center gap is subjected to the least amount of stress. Furthermore, the thermal resistance of the SAC305 solder layer also increases with the void ratio. It is inevitable that the flip-chip LED package produces voids during reflow soldering. The next step we need to perform is to optimize the reflow soldering curve to reduce the possibility of voids and enhance the material’s shear resistance. According to the influence of void ratio on the mechanical and thermal properties of the package, a method to optimize the solder layer is proposed. In addition, the flip-chip packaging of the LED has greater advantages in high-power and integrated packaging and needs to be strengthened in low-power and medium-power applications.

### 2.2. 3D Package and TSV

**Figure 4:**
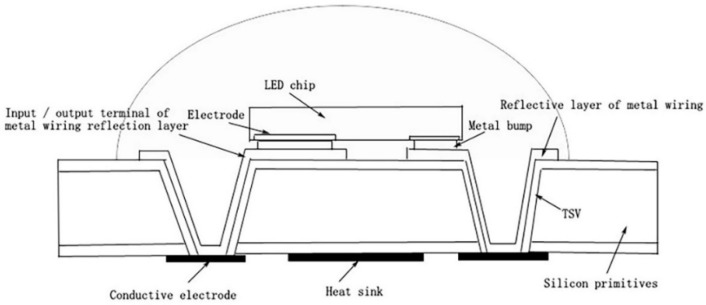
Diagram of a low thermal resistance wafer-level LED package [33].

Affected by flip-chip LED packaging, vertical packaging and chip integration, 3D packaging and through-silicon via (TSV) technology emerged. The 3D package [29] is stacked one by one with the integrated circuit chips and interconnected vertically, which makes the package smaller, lighter and posesses improved heat dissipation [30]. The core of the 3D package is obtained by through-silicon via (TSV) technology [31]. Unlike traditional chip packaging, TSV realizes the electrical interconnection between the chips on a three-dimensional level, which greatly increases the package’s density and its vertical interconnection. Furthermore, it improves the signal transmission speed between the chips and reduces the power consumption of the chip. The combination of the 3D package and TSV technology creates efficient thermal management and is primarily used for high-power LEDs. Chen et al. [32] applied TSV to wafer level packages, which not only reduces signal delay and loss but also increases the signal transmission speed and allows the heat generated in the chip to transfer directly through the silicon pass. The hole is discharged, and heat dissipation efficiency is enhanced by 30% compared to a conventional wafer-level package. Zhang et al. [33] designed a low thermal resistance wafer-level LED package (Figure 4). The structure mainly includes LED chips, metal bumps and TSVs. The LED chip is placed on a metal bump on the front side of the silicon base body and a thermally separated motor assembly composed of conductive electrodes and a heat sink is placed on the back side. The TSV is set outside the area perpendicular to the LED chip and the chip electrode passes through the TSV via metal wiring. The reflective layer is in electrical communication with the conductive electrode. This structure facilitates the heat dissipation of the LED chip and improves the heat dissipation of the LED chip relative to the external pins of the package. Additionally, it significantly reduces the thermal resistance of the package’s structure.

Not only can the addition of TSV technology to wafer-level packaging shorten the heat dissipation path, but it can also integrate with the optical design of the LED package; promote the conversion of the phosphor layer; take away the heat released from the chip to the phosphor layer; and improve the reliability of the phosphor layer. The packaging method that develops a hermetic wafer level package for a LED module with a phosphorescent ceramic converter relies on the silicon interposer wafer of the TSVs that bonds the gold source using seal rings and binds it to the silicon frame wafer to constitute a cavity. After the LED is bonded to the silicon wafer, the cavity containing the LED is sealed by a phosphor conversion ceramic. The frame wafer has through-holes with inclined mirrored side walls to allow light to be optimally reflected, which provides the mounted LED high efficiency. In this new architecture, the ceramic converter is not directly connected to the LED chip but to a part of the package, and the distance from the LED is fixed. This enables the heat generated by light conversion to be dissipated directly into the package through the ceramic converter rather than through the LED itself. As a result, the LED chip is not affected by heat, and the service life of the device is significantly extended [34].

However, if the heat generated between the chips is not removed in time during the stacking process, it affects the reliability of the device. This is the most significant problem to be solved in 3D packaging. In addition, TSV technology can be used for substrates and other materials for packaging, but the through-holes formed by this technology fail due to the high thermal expansion mismatch between the two different materials after being filled [35].

### 2.3. Ultra-Thin Package

**Figure 5:**
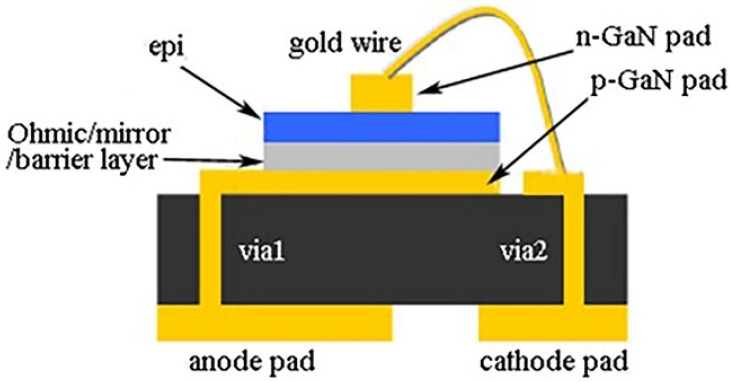
Schematic diagram of TFP LED structure [43].

The flip-chip as well as the 3D and TSV technology have evolved, since electronic packages have become thinner and smaller [36]. Even the lead in the package is designed to provide less space upon contact, and the pitch and width of the wires are appropriately reduced, which decreases the size of the package [37]. For example, an ultra-thin package without leads makes the coating of phosphor easier and reduces the amount of phosphor used. Additionally, there is no risk in damaging the gold wire with the phosphor coating, which makes the package thinner. Kleff et al. [38] developed a new ultra-thin, high-brightness LED package for wafer-level packaging that includes a vertical LED chip package. Before inserting the LED, it is necessary to plate two layers of copper on the glass including a 10 μm copper pillar for the close contact of the back of the LED with other materials. After embedding the LED, a sealing material, namely benzocyclobutene (BCB), is deposited on the back side of the LED. The BCB layer and the copper-plated track are opened to create a point connection on both back sides. This packaging does not use wire bonding so that the structure is very reliable. In addition, an advanced ultra-thin flexible LED (FLED) packaging technology that reduces the thickness by 82.7% compared to COB and is 35% thinner than Panasonic Organic Light-Emitting Diode (OLED) lighting [39]. While the package is thinner, heat dissipation also needs further attention. Ultra-thin packages can be used as the overall package of optoelectronic devices and for specific packages such as WLP and CSP. The CSP package is defined as a chip-level package. The area of the CSP package is no more than 120% that of the chip [40]. The distance between the metal substrate and the heat sink is short, which greatly improves the reliability of the memory chip after long-term operation. Line impedance is significantly reduced, which also makes the chip run faster. Compared with the ball grid array package (BGA), the CSP package increases storage capacity by three times in the same space. Therefore, CSP packaging has received extensive attention in the electronics packaging industry [41]. Huang et al. [42] combined direct illumination backlight to design a free-form design chip-scale package and produce a suitable mini-LED. The freeform-designed chip scale package (FDCSP) method effectively reduces the size of the package and enhances heat dissipation. Wen et al. [43] used silicon-based packaging to mount a vertical thin-film LED directly on the package base to form a novel thin-film LED package (TFP), as illustrated in Figure 5, and achieve higher requirements for vertical LEDs. In order to form a vertical current injection, the layered LED epitaxial thin-film is directly bonded to the package substrate via a P-GaN pad. Then, the n-GaN pad is bonded to the pad of the substrate by wire bonding. Compared to conventional thin-GaN LED, this structure eliminates the chip carrier and the first bonding layer and minimizes the thermal path from the LED junction to the base. Therefore, although the ultra-thin packaging technology reduces the thickness of the package and enhances heat dissipation by reducing the thickness of the package or removing the corresponding bonding layer, the changes in the reliability of the package while reducing the thickness should be payed attention to.

## 3. Package Structure

There are three major heat dissipation paths: (1) chip → phosphor layer → silicone lens → environment; (2) chip → gold wire → electrode pin → environment; and (3) chip → thermal interface material (TIM) → heat sink substrate → heat sink → environment; however, they are limited. We can innovate the structure of the package, alter the material with a higher thermal conductivity or add an external heat sink to improve heat dissipation. The design of the package substrate, lens and phosphor layer has a significant impact on heat dissipation. Herein, we summarize how to enhance heat dissipation inside the LED package by developing packaging structures and new packaging materials.

### 3.1. Substrate Design

**Figure 6:**
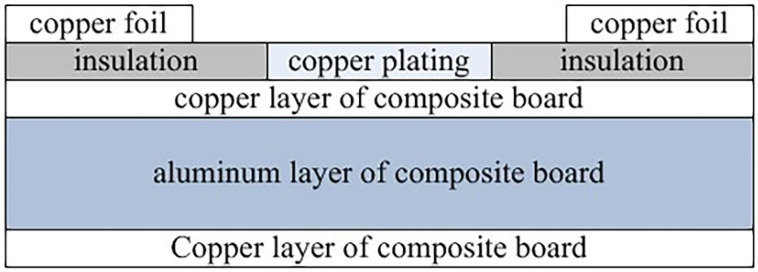
Schematic diagram of composite metal plate.

The package substrate plays the role of a bridge in package. The most important thing is the case that the package substrate is the main thermal channel for the heat of the LED chip in the high-power LED device package to diffuse into the environment. The substrate material of the heat dissipation must have high electrical insulation performance, high stability and high thermal conductivity, and its thermal expansion coefficient, flatness and high strength are consistent with the chip [44]. The substrates of LED packaging have mainly experienced ceramics (Al_2_O_3_, AlN and SiC) [45,46], silicon [47], glass [48] and metal substrates. Metal substrates include Cu substrates and Al substrates [48,49]. Metal substrates have stronger thermal conductivity than non-metal substrates. However, when using a metal substrate as an LED package substrate, its conductivity needs to be considered. In consideration of electrical safety, the metal substrate must be made of insulating materials or coated with an insulating film to prevent short circuits and leakage caused by conductive contact with the substrate. The ceramic substrate itself is an insulator, and there is no need to add an insulating layer [50]. The Al substrate and Cu substrate covered with an insulating layer can still achieve strong heat dissipation, but simply using the Cu substrate to gain heat dissipation increases the cost of the package. Therefore, the metal composite substrate was produced under this situation. Huang et al. [51] produced composite substrates of copper-aluminum by anodizing the upper and lower sides of the aluminum plate and then using high-temperature explosive compounding or sputtering (as shown in Figure 6).

The composite substrate of the copper-aluminum-copper is made by high-temperature explosion in that the sputtering method is relatively expensive. The results show that its heat dissipation effect is the same as that of Cu, and the change to the composite board solves the problem that aluminum cannot be directly welded. In addition, the heat generated by the chip can “bypass” the high thermal resistance insulating layer after the copper-clad laminate has undergone “primary milling” and “secondary milling.”

Compared with the traditional heat dissipation substrate, the heat dissipation channel reduces an insulating layer in the package. With the use of higher forward current and the use of multi-chip packages to increase the total light output, the junction temperature of the LED package rises due to the accumulation of heat, resulting in a lot of heat under the die. In this case, the chip on board (COB) can be combined with the power electronic substrate to enhance the overall heat dissipation effect of the package [52]. Apart from using a metal substrate, the thermal management performance is also able to be improved by coating a silver layer on a glass substrate or coating graphene on other non-metal substrates. Wu et al. [53] deposited a diamond-like carbon film on the packaging substrate of light-emitting diode; exploited an infrared thermal imager to study the influence of the thickness of the diamond-like film on heat dissipation performance; and utilized finite element analysis to conduct thermal simulations on the diamond-like carbon film light-emitting diodes with different thicknesses. The result of the experiment revealed that the surface temperatures of LEDs with the thicknesses of 1.6 μm, 2.4 μm and 3.2 μm diamond-like films on aluminum substrates are 1.2 °C, 1.5 °C and 2.3 °C lower than those without diamond-like films. The simulation results are consistent with the experimental results, indicating that a thicker diamond-like carbon film has a better effect on improving the heat radiation of the substrate.

In addition to changing the material composition of the substrate to enhance the heat dissipation effect of the heat dissipation substrate itself, a heat dissipation hole can be provided on the substrate. Xu et al. [54] raised a structure of the ceramic substrate with copper-filled thermal holes to improve the thermal management and lifetime of DUV-LEDs. The thermal resistance of 4 × 4 thermal holes based on DUV-LED is reduced by 23.04% compared with the traditional structure. While the heat dissipation holes dissipate heat, they will also affect the thermal expansion mismatch between materials. However, there is an exception that the heat dissipation path can also be shortened by removing the substrate. Nie et al. [55] proposed a novel packaging structure of chip-on-package (COB), which is mainly composed of six parts: lens, phosphor, chip, plastic layer, adhesive layer and heat sink. This structure focuses on removing the substrate on the basis of the common of COB with a large number of layer structures. The LED chip is embedded in the plastic layer by a pre-plastic film packaging method in the package [56], and then it is sealed with plastic phosphor. The heat generated by the LED chip is dissipated to the outside through the directly connected heat sink, which not only shortens the cooling path but also reduces the junction temperature. In addition, the fewer materials used in the process, the fewer quality problems will occur. One of the reasons for this situation is that different materials have different thermal expansion coefficients. Removing the substrate reduces the cost, paying attention to lateral and vertical heat transfers between the chip and the plastic material is required as well.

### 3.2. Lens Design

From all structures and materials that do not emit light, the dome lens has the most amount of influence on light output [57]. Convex lenses, concave lenses, spherical mirrors, microlenses and free-form lenses have successively been used [58]. Some studies present improvements on the structure of the lenses, while others focus on the materials used.

**Figure 7:**
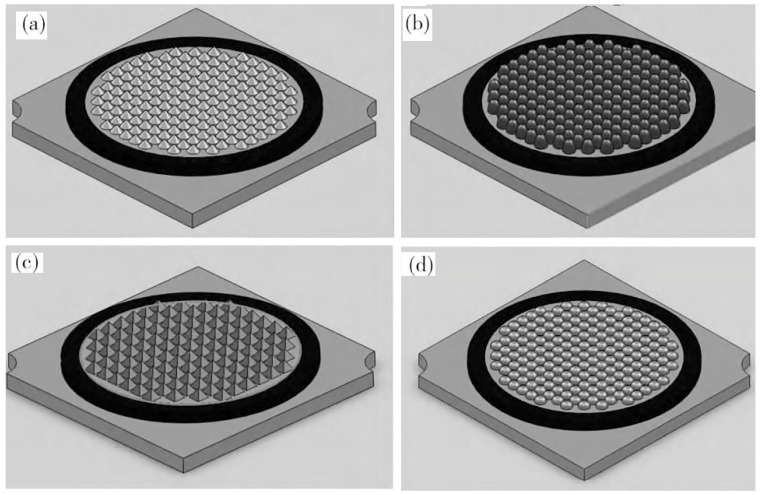
Three-dimensional rendering of the models for the lens of packaged LEDs: (**a**) array cone lens, (**b**) semi-ellipsoidal lens, (**c**) quadrangular pyramid lens and (**d**) semi-spherical lens [62].

Yu et al. [59] made a glass blister on the wafer package by using a chemical foaming process (CFP) instead of an epoxy resin to solve the issue of the yellowing of the epoxy resin from long-term heating and radiation and the reduction in light output over time. Such issues also shorten the lifetime of the LED. Glass has excellent thermal and physical properties compared to epoxy resins. The glass bubble cap made by CFP effectively improved the reliability and optical performance of a white LED. A small amount of foaming agent foam is required to prepare a medium spherical glass cavity. However, the size of the glass cavity cannot be precisely controlled. Zou et al. [60] studied the chemical foaming process by preparing a glass cavity with a hemispherical shape and encapsulating it by using a micro-glass bubble array combined with a silicon substrate. The improved chemical foaming process solves the cost of dry etching and offers better control over the shape of the bubble array. The glass ball is a hemisphere, which reduces the volume of the package and effectively improves the luminous efficiency of the LED. In addition, the package can also combine the lens with a silicone sealant to reduce the amount used. Compared to conventional lens packages, the volume of the lens decreased, which reduces the reflectivity of the lens [61]. Half lenses have also been shown to be used on COBs. Lin et al. [62] introduced array cone lenses, semi-ellipsoid lenses, quadrangular pyramid lenses and semi-sphere lenses into the packages of 1919 COB LEDs in optical simulations. The four structures are shown in Figure 7. Their results indicated that the light extraction efficiency of the LED package with a 0.5 mm high array cone lens with a diameter of 0.9 mm was 25.8% higher than that of planar packages. In addition, the hemispherical lens package had 18.8% higher light extraction rate than the planar structure. This illustrates that the structure of the red-green-blue (RGB) COB-LED can significantly improve light extraction rates.

Furthermore, Lee et al. [39] designed a micro-lens array for LEDs used in indoor illumination to meet the requirements of flexible packaging, enhance the strength of the LED strip and improve its portability. They designed a micro-lens for panel illumination that was passed through ultra-thin flexible LEDs (FLED) and molded on the package. The design has a flexibility similar to that of an organic light-emitting diode (OLED) flexibility, but it has higher luminous efficiency. In short, the design of lenses currently follows strong luminous efficiency, while avoiding the use of materials with strong reflection to ensure the timely dissipation of heat inside the package.

### 3.3. Phosphor Design

Currently, phosphor packages essentially use a remote phosphor package [63]. The phosphor in the package is far away from the chip, which reduces the heat produced by the chip. Accordingly, the temperature of the phosphor layer is decreased, and light efficiency and stability are increased [64]. In addition, Liu et al. [65] showed that remote phosphors provide better light extraction, but the distance between the phosphor and the chip is restricted. If the distance is too high, light extraction efficiency is reduced. Kim et al. [66] studied the thermal behavior of a remote diode by monitoring the distance between the phosphor layer and the substrate. They determined an optimal phosphor layer distance of 320 μm for a remote LED package. The light output of the optimal position was 6% higher than when the phosphor layer directly covers the LED chip.

**Figure 8:**
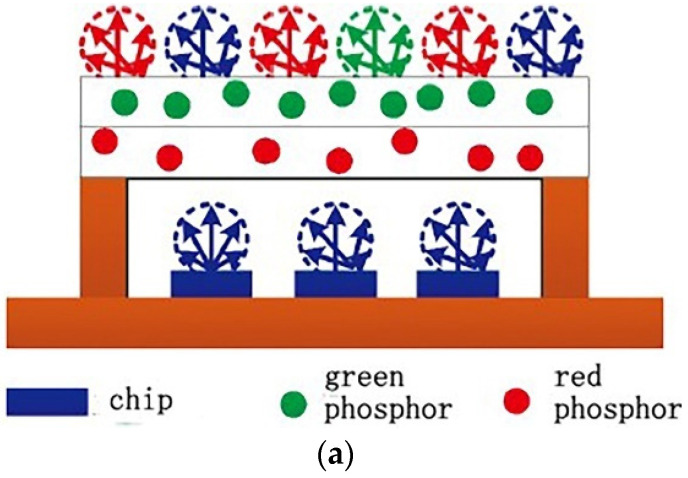

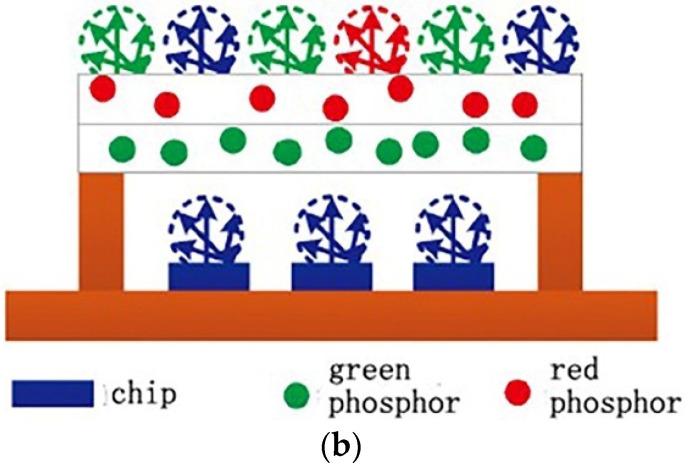
(**a**) Blue-red-green (B-R-G) and (**b**) blue-green-red (B-G-R) film packages [67].

Light efficiency is not only related to the distance between the phosphor and the chip but also to the shape and the distribution of the phosphor layer. One phosphor color is not enough to improve the brightness of white light emitting diodes (WLEDs). However, when multiple phosphors are directly mixed, it involves different compounds. Mixing two different phosphors directly triggers reabsorption between different phosphors, which results in energy loss. Zhuo et al. [67] used phosphor layering and a remote fluorescent packaging to prepare a double-layer film by hot-pressing. The effects of the different lamination orders and the different emission wavelengths of the green and red remote fluorescent films on the spectral performance of white LEDs were studied using a fluorescence spectrophotometer and a spectrum analyzer. They found that the blue-green-red (B-G-R) film packaging improved radiation luminous efficiency by 31.69% compared to a blue-red-green (B-R-G) film. Additionally, the fidelity of the color and the gamut index both increased with the wavelength of the red remote fluorescent film, whereas fidelity gradually decreased when the wavelength of the green remote fluorescent film increased. The gamut index first decreased and then increased. Figure 8 shows the blue-red-green (B-R-G) and the blue-green-red (B-G-R) film packages. The combination of micro-lens and phosphor can be made into a 3-D micro-lens phosphor with curvature. This structure installs a hemispherical micro-lens array on the upper layer of the phosphor. Photons avoid internal reflection and escape through the lens surface with high curvature so that a large amount of random light can be emitted. Moreover, the hemispherical structure enlarges the contact area between the phosphor and the air, which helps the package’s heat dissipation [68].

The designs of stacked structure and the segmented help understand the position and proportion of the blue and red chips better to control the color temperature of the device. The stack solves the issues of overlapping green and red spectra, which improves the pure color of the device. The amount of red and green phosphors used was reduced by mixing the phosphors in the structure [69]. However, during long-term use, traditional phosphors absorb many wavelengths when they emit light due to their large half-width. This reduces the illumination efficiency of the device and affects its color. The coverage area of the domain is not expansive enough. Quantum dots (QD) are nanoscale semiconductor luminescent materials that generally have a particle size between 1 and 10 nm [70]. LEDs based on multicolor quantum dot conversion enable a color rendering index of Ra > 95 and R9 > 95 [71]. Therefore, quantum dots are becoming heavily used as packaging materials. Li et al. [72] put forward the application of quantum dot (QD)-phosphorescent hybrid structure in high-efficiency light-emitting diodes. By studying two separate package structures (SPSs), the green-QD-down and red-phosphor-down, the green-QD-down SPS exhibits higher backscattered loss while exhibiting less conversion loss. Moreover, the green-QD-down SPS exhibits better heat dissipation of QDs by reducing the heat path from QDs to the lead frame, as well as less thermal power generation in conversion layers. The high operating temperature of the light-emitting polymer layer with low thermal conductivity challenges the thermal stability of quantum dots. Zhou et al. [73] used an ice template to fill the light-emitting layer with hexagonal boron nitride sheets (hBNS) arranged vertically to enhance the vertical thermal conductivity of the light-emitting layer. This creates a relatively high thermal conductivity heat dissipation channel for luminescent particles. The proposed new vertical enhanced quantum diode package design is compared with the thermal performance of isotropically enhanced quantum diodes (Iso-WLEDs) and ordinary quantum diodes (Com-WLEDs). It is found that the maximum operating temperature of the direct enhanced quantum diode package under the same operating current is the lowest. The new method of vertical thermal conductivity of the light-emitting polymer layer has certain guiding significance for the application of quantum dots in high-power light-emitting diodes.

## 4. Conclusions

Heat dissipation of LED packaging has currently been a hot spot of research. Flip-chip LED packaging, 3D packaging and ultra-thin packaging technology all mean that LED packaging is developing in the direction of thin, small and strong heat dissipation. These three packaging technologies have their own advantages in the heat dissipation of the package. However, the reliability of the package structure also needs to be guaranteed when using the technology. This relates to the effect of reflow soldering of flip-chip package solder joints and the voids generated by the solder joints on the thermal and mechanical properties of the package; thermal mismatch between materials and the impact of TSV radius on TSV; and the impact of ultra-thin packaging on the service life of the device. In addition, with the development of high-power devices, the design of the substrate, lens and phosphor layer of the LED package attracts the most attention. As a bridge between the chip and the environment, the substrate plays a major role in packaging. However, a substrate with high thermal conductivity may not be able to achieve a high heat dissipation effect. It is a critical value that the thermal expansion between different materials on the substrate exerts force on the package. The light transmittance of the lens, high-efficiency light extraction and the position and material of the phosphor layer are also the most important research topics. Although the quantum dots used in the phosphor layer have been widely used for packaging, their performance is unstable. Temperature, air and water have a great influence on the performance of the quantum dots and the light efficiency of the LED chip, which requires further research. This review mainly describes the development of LED packaging technology and related components in terms of heat dissipation performance, but there are still many undiscovered aspects.
